# Sexual and reproductive health focus group discussions with Syrian men and women living as refugees in the Bekaa Valley, Lebanon

**DOI:** 10.1007/s44155-024-00089-x

**Published:** 2024-09-10

**Authors:** Anna C. T. Gordon, Loubaba Mamluk

**Affiliations:** 1 Elizabeth Blackwell Institute, University of Bristol, Bristol, UK; 2 Bristol Medical School, National Institute for Health Research (NIHR) Applied Research Collaboration (ARC) West, Population Health Sciences, University of Bristol, Bristol, UK

**Keywords:** Refugees, Sexual health, Reproductive health, Refugee camps, Lebanon, Syria

## Abstract

**Background:**

Conflict in Syria since 2011 led to over one million Syrians fleeing to Lebanon, predominantly from economically disadvantaged areas with low literacy and high child marriage rates. Over 90% live in extreme poverty, in informal tented settlements with minimal access to education, healthcare or employment. Displacement and poverty have further increased early marriages and unplanned pregnancies, and curtailed access to sexual and reproductive healthcare (SRH) in the Bekaa valley. This is exacerbated by increasing rates of sexual and gender-based violence (SGBV), intimate partner violence and domestic violence.

**Study design:**

We aimed to explore SRH beliefs and practices and teach on key SRH topics through focus group discussions (FGD) and questionnaires, co-designed with Syrian practitioners, conducted with Syrian men and women. FGD were recorded, transcribed and thematically analysed. Questionnaires collected demographics and explored SRH beliefs and practices.

**Findings:**

24 FGD with 203 participants, 72.4% female and 27.6% men. 90.1% participants were married with an average age-gap of 6.3 years between partners. Teenage marriage rates were 55.6% for women, and 47.4% delivered their first child before the age of 20. 43.6% participants were not using any contraception. Findings demonstrate the impacts of SRH cultural norms and changes due to displacement, financial crisis, and increased exposure to technology and NGOs.

**Conclusions:**

SRH is multifaceted and contested, requiring systemic improvements in access to care, employment and education. This small but important proof-of-concept study demonstrates the possibility of engaging men and women in SRH discussion; paramount to empowering communities and challenging intergenerational SGBV.

## Introduction

The Bekaa valley lies 15 km from the mountainous Syrian border and has been home to over 300,000 Syrian people since the start of the war in 2011 which forcibly displaced over 6.8 million people, half of whom are children [1]. Lebanon was not a signatory of the 1951 United Nations Refugee Convention, and Syrians live under strict regulations restricting their mobility and ability to work, whilst experiencing a plethora of barriers in accessing scant healthcare and education [2–4]. There is escalating tension between the Syrian and local Lebanese populations centered around resource scarcity and deteriorating security acutely exacerbated by the Lebanese economic crisis which began in October 2019 [5]. The crisis ranks amongst the worst globally since the mid-nineteenth century, forcing over 80% of the national population into poverty, and 90% Syrians in Lebanon into extreme poverty [1, 4–8]. The Syrian population in the Bekaa have lived in informal tented settlements (ITS) on privately rented land supported by aid agencies for over ten years, often lacking clean drinking water, secure electricity supply or sustainable infrastructure [1, 4].

ITSs range from small family groups to over 100 tents with populations from across Syria, and an ITS community-selected leader assumes responsibility for managing aid provision and social matters in each one. Residents report receiving more aid if they select a female leader, therefore despite the population’s patriarchal cultural norms, many ITS have nominated female leaders.

Syrian refugees displaced to Lebanon have predominantly lower educational attainment and come from more disadvantaged socioeconomic strata than the national Syrian pre-war average, or Syrian populations in other countries. This contributes to a high baseline in rates of teenage marriages, births and cousin marriages (viewed traditionally as protective as suitor’s family background and loyalty are known) compared to Syrian refugee populations elsewhere [8–11]. Poverty and political vulnerability specific to Lebanon has further increased rates of teenage marriage, but decreased birth rates [8, 9, 11].

Despite this, in multiple previous studies from the Bekaa, male and female participants unanimously expressed opposition to teenage marriage, but highly gendered narratives exist surrounding perceived factors necessitating the practice [10–15]. Men have cited economic instability or safety as primary drivers; some fathers of young girls arranging for them to be married repeatedly in short-term contractual marriages exclusively for sex to alleviate their family's poverty through bride price payments. Others discussed the need for early marriage to protect women from violent sexual assaults by Lebanese men, particularly women employed as domestic staff [13, 14].

Women also discuss fears of harassment and the need for protection of “al sutra”; a girl's honor, from increasing rates of harassment perceived to be predominantly from Lebanese men [11, 13, 16–19]. High levels of harassment have forcibly restricted women’s mobility and thus their access to education and healthcare, causing both mothers and young women to cite harassment and frustration at their restricted mobility as drivers of early marriage [10, 11, 20, 21]. Further, women report ongoing harassment and discrimination within education and healthcare facilities from professionals, compounding disruption and curtailment in health and education already experienced through displacement [12, 21–23].

Limitations in access to healthcare and education impact directly upon sexual and reproductive health (SRH) with contraception usage and attendance at antenatal appointments significantly below pre-war rates in Syria [10, 16, 17, 21]. Lack of safe transport, resources and hostility has increased premature school drop-out rates, limiting access to SRH education outside of the home for young people as young adults would normally at least learn about reproduction in biology in the eighth grade [12, 24]. Young women are therefore actively disempowered by early marriage and left dependent on their mothers or husbands for information deemed extremely culturally sensitive regarding SRH, such that they often receive none at all until their wedding night, or are presented with misinformation and myths [25, 26].

Though both genders’ narratives around early marriage focus on the need to protect women in the public sphere, in private spheres there is widely reported high and increasing rates of intimate partner violence (IPV) and domestic violence (DV) intensified by displacement, poverty and an increased age-gap between partners [14, 19, 21, 27]. To protect the family honor, the threat presented by harassment or assault is exchanged for the certainty of sexual and gender-based violence (SGBV) inherently presented by perceived cultural norm of child marriage. This is paired with the rising, but less visible and publicly shameful risk of domestic violence (DV) or intimate partner violence (IPV) [14, 16]. Cultural norms of high birth rates, low SRH literacy and early marriage in this setting, motivated by the patriarchal honor-based culture serve to propagate intergenerational cycles of violence against women [13, 15].

In this study, we partnered with a well-established grassroots medical non-governmental organization (NGO) conducting mobile clinics into ITSs in the Bekaa, and co-designed a project based on their clinical priorities; teenage pregnancies and obstetric complications. The NGO, “Endless Medical Advantage” (EMA) is run by Syrian clinicians and has excellent rapport and established relationships with the Syrian population and thorough understanding of the political and cultural context. During study conceptualization both EMA, and that research team felt strongly that the research should offer individuals some benefit in return for participation. We lacked the ability to provide financial incentive, but together agreed that delivering culturally sensitive teaching following focus group discussions (FGD) on key topics covered could empower the study population by addressing significant current knowledge deficits potentially driving the clinical issues outlined above. We therefore co-designed focus discussion group (FGD) sessions for women and men with the dual purpose of first aiming to explore SRH beliefs and practices, and then to deliver teaching on key SRH topics. This paper presents results from these mixed format education and discussion FGD sessions.

## Methods

### Study design

This was a predominantly qualitative study, phenomenological in nature, collaboratively designed and conducted with EMA from conceptualization to delivery. Our dual aims were; firstly, to use grounded theory to explore the SRH beliefs, and secondly, following this to provide accurate and culturally sensitive and specific SRH education in a setting where this is tabooed, without exposing individuals to stigmatization [28, 29]. We used FGDs to facilitate discussion of SRH beliefs and practices at a population level, providing a natural platform from which to deliver specific educational material guided by participants' priorities. A confidential questionnaire outlining participants’ demographic information and personal SRH beliefs and literacy was collected after each session. Questionnaires were completed where limited literacy necessitated with assistance from our interpreter, but participants were encouraged to keep responses confidential from each other.

Study materials were designed collaboratively remotely, and then in-person when AG arrived in Lebanon in August 2022, with the cultural and methodological expertise of LM (a Syrian epidemiologist) and EMA’s setting specific knowledge. Standardized toolkits were used, including the Inter-Agency Working Group of Reproductive Health in Crises (IAWG) field manual and instruments designed by the inter-agency Human Reproduction Program (HRP) aided by a scoping literature review [30, 31]. Ethical permission was granted by the University of Bristol Faculty of Health Sciences Research Ethics Committee.

### Sampling and recruitment

Once in Lebanon in August 2022, up to two weeks prior to a proposed session, AG and EMA staff approached leaders of local ITS’ with whom they had an established relationship and discussed the contents of the proposed session. AG was presented as a British doctor conducting research, working associated with EMA. She responded to any questions. The ITS leader then purposively selected and approached individuals, face-to-face, in their ITS whom they felt appropriate to invite to sessions based on age, availability and marital status. Participation of all married and unmarried males and females above the age of 16 was welcomed by the EMA team but left to the discretion of each community [32].

### Data collection

FGD were conducted in August and September 2022 by AG, a female British doctor with basic Arabic skills, and previous qualitative research experience with vulnerable populations and sensitive topics. We used a professional local female Lebanese interpreter who was specifically trained prior to the first session. She interpreted verbatim everything spoken by all participants, though where there were extended periods of group discussion, contributions made by each participant were summarized in English. Sessions were audio recorded using encrypted devices.

FGD ranged from 6 to 18 participants aged between 16 and 68, hosted in private in family tents usually belonging to the leader to ensure content shared was kept confidential. Prior to commencing sessions, the purpose and proposed content was explained to all participants and verbal consent gained. Participants were informed that they were free to leave at any time, withhold information they were uncomfortable discussing and were encouraged to discuss generalized cultural norms, rather than personal behaviors or experiences to minimize stigma around the highly sensitive topics. The consent process was repeated prior to questionnaire distribution.

Sessions content began with more straightforward topics and building to more sensitive content and is outlined in Appendix 1. Information given by AG was based on the World Health Organisation (WHO) guidance, and presented as a “medical perspective”, culturally non-specific, and based around global medical statistics and risks. Sessions were piloted and then conducted iteratively with time spent discussing topics led by participants. AG kept a reflective diary, including notes on non-verbal communication, and degree of homogeneity in opinions (later termed ‘consensus’) around key discussions topics in each group after each session in collaboration with the interpreter (Table 3).

Sessions were continued to our target of 200 participants, within which data saturation was achieved.

### Data analysis

The accuracy and quality of content interpreted was checked by LM, a native Levantine Arabic speaker before recordings were transcribed verbatim in English. All participants’ names or identifiable information were removed during transcription. Qualitative data were thematically analyzed using NVivo 12 [33, 34]. AG and LM coded the data with regular analysis meetings. Themes were iteratively derived entirely from the data [35, 36]. It was not possible to approach participants with either recordings, transcripts, or for feedback on findings, due to new practical and security constraints in accessing the ITSs since the study began.

Quantitative questionnaire data were analyzed using STATA and simple summary statistics generated, with linear regression or unpaired T tests where relevant.

## Results

### Demographics

We conducted 24 sessions; 16 women's sessions, and eight men’s sessions in a total of 16 ITSs. Nine ITSs were led by men and seven by women (Table 3). Sessions were attended by 203 participants, 72.4% female, and 27.6%, 90% of whom were married (Table 1).

**Table 1:** Demographics of study population including marital status, age and family structures

	Male n = 56 (27.59%)	Female n = 147 (72.41%)
Age	Range: 17–68 Mean 39.56 (12.43)	Range 16–68 Mean 32.97 (11.3)
Literacy (Y/N self-declared)	n = 52 31 (59.62%)	n = 146 89 (60.69%)
Years of schooling	Range 0–15 Mean 6.88 (3.66)	Range 0–20 Mean 5.48 (3.71)
Employed	n = 56 28 (50%)	n = 142 59 (41.55%)
Siblings	Range 0–22 Mean 8.66 (3.7)	Range 0–21 Mean 8.3 (3.35)
*Marital state* Married Single Divorced Widowed	n = 56 50 (89.29%) 6 (10.71%) 0 0	n = 146 132 (90.41%) 1 (0.68%) 6 (4.11%) 7 (4.79%)
Age of marriage	Range 15–49 Mean 25.02 (5.77)	Range 12–36 Mean 18.43 (4.10)
Age at birth of first child	Range 19–42 Mean 27.0 (4.96)	Range 13–39 Mean 19.88 (4.1)
Number of children	Range- 0–11 Mean 4.17 (2.35)	

**Fig. 1:**
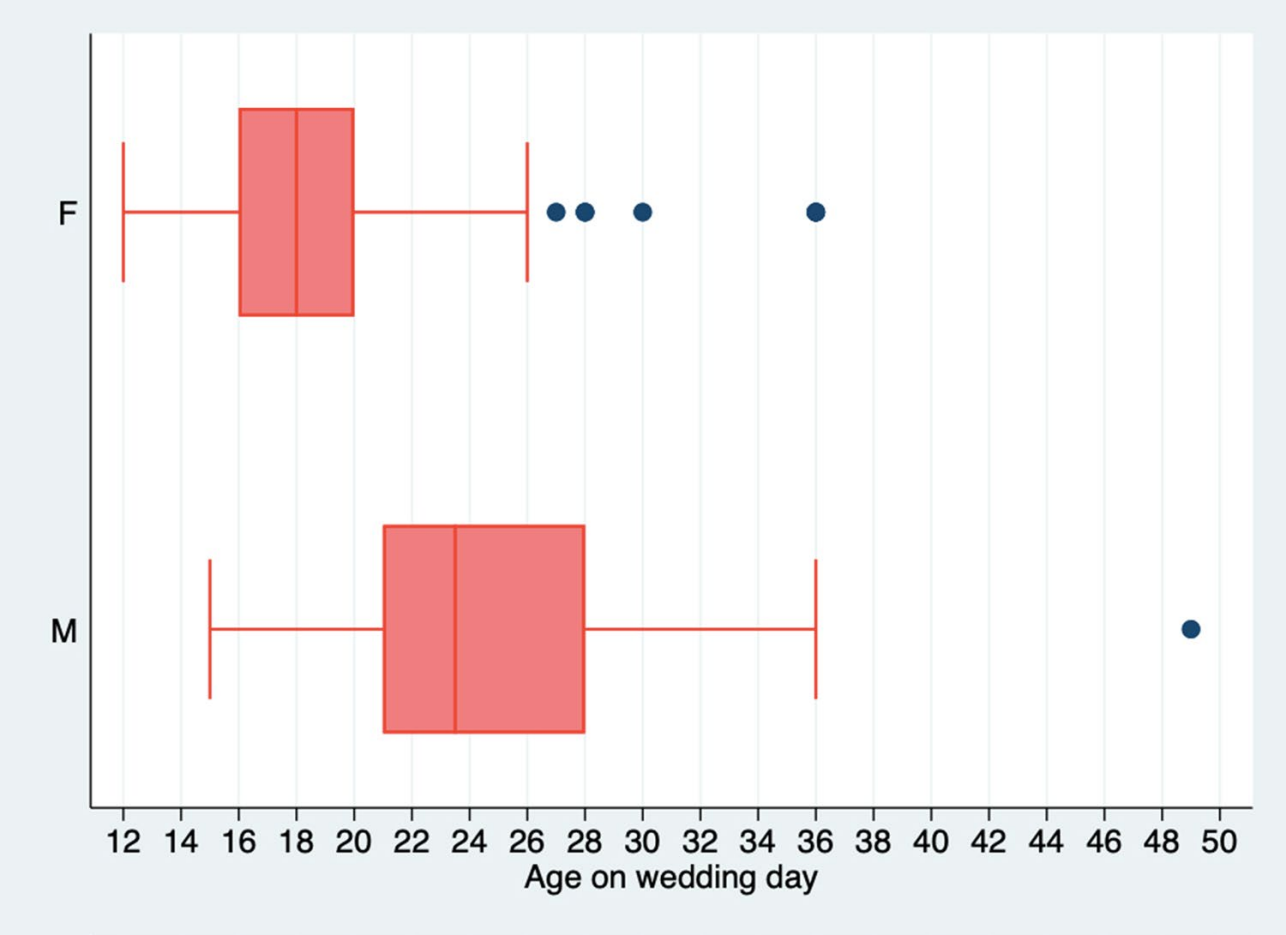
Box and Whisker plot showing age on wedding day of men and women

Overall, 8% of men and 63.4% of women were married as teenagers (55.6% women, and 4% men under 18 years old), and 47.4% women delivered their first child before the age of 20. The mean reported age-gap between partners was 6.3 years (SD 6.44), the largest was 35 years (Table 1, Fig. 1).

**Fig. 2:**
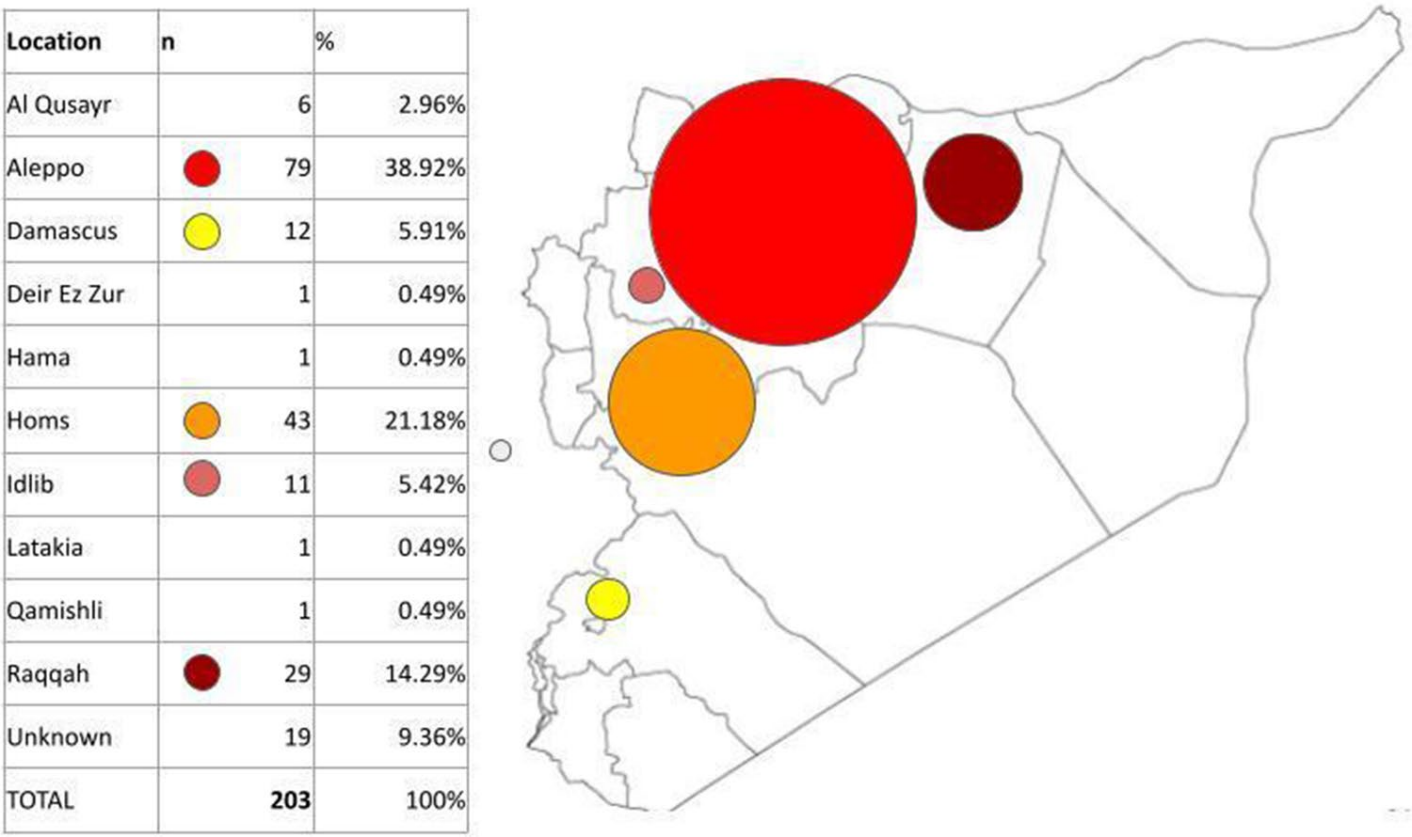
Map showing participants region of origin (city vs rural not differentiated)

Participants originated from rural and urban areas across Syria, predominantly small settlements in the countryside around Aleppo and Homs (Fig. 2). Men had attended a mean of 1.4 years more education than women (95% CI 0.8–2.6, SE 0.6) and 15% of women, and 8% men had never attended school. Average years of schooling did not vary significantly between participants of different ages.

### Reproductive health literacy

Contraception was predominantly believed to be a couple's decision; 74.3% participants answered that they had discussed contraception with their partner, 76.4% that contraception was a “joint” decision, and 83.1% answering that it was a “joint” responsibility. However, 15.9% of men and 11.9% of women felt that contraception was exclusively a “man's” decision, whereas no men, and only 11.9% women felt it was exclusively a woman’s decision.

**Fig. 3:**
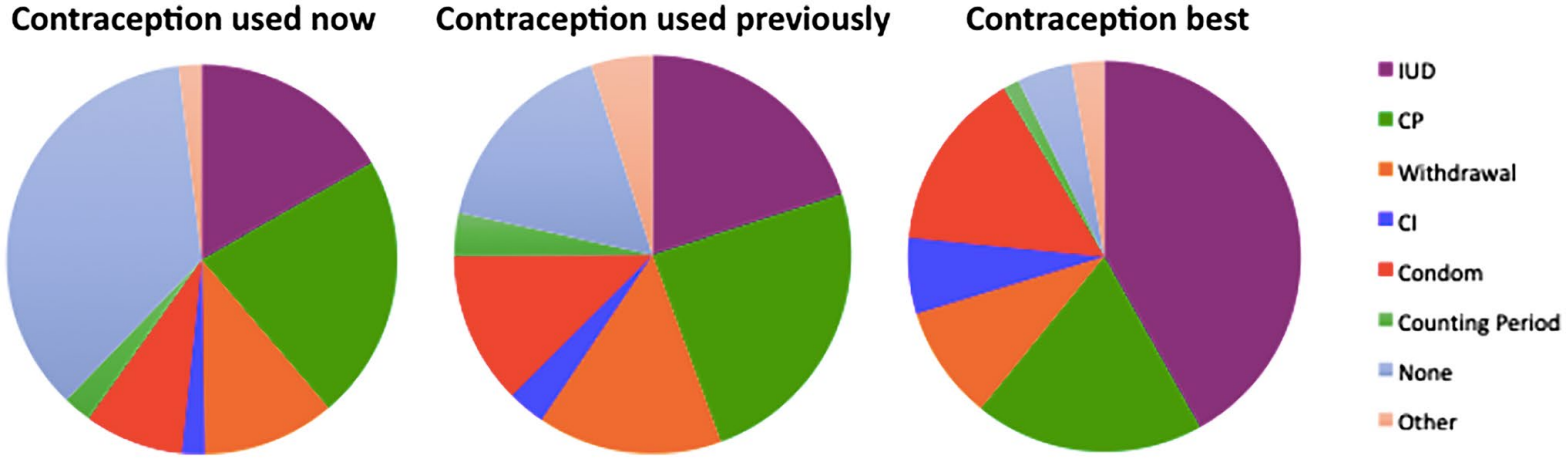
Pie charts demonstrating previous usage of contraceptives (unspecified timeframe), current use, and contraceptive method considered ‘best’, demonstrating gaps in provision of acceptable contraception in Lebanon

The contraceptive pills (CP) and intrauterine devices (IUDs) were the most widely known, available and favoured forms of contraception amongst both men and women (Fig. 3). Though 80.4% of participants answered that contraceptives were available to them, 43.6% were not currently using any. 24.4% participants had never used any form of contraception, 81.8% of participants had never used a condom (Fig. 3).

### Qualitative findings

FDG qualitative findings were arranged into themes and subthemes (Table 2).

**Table 2:**
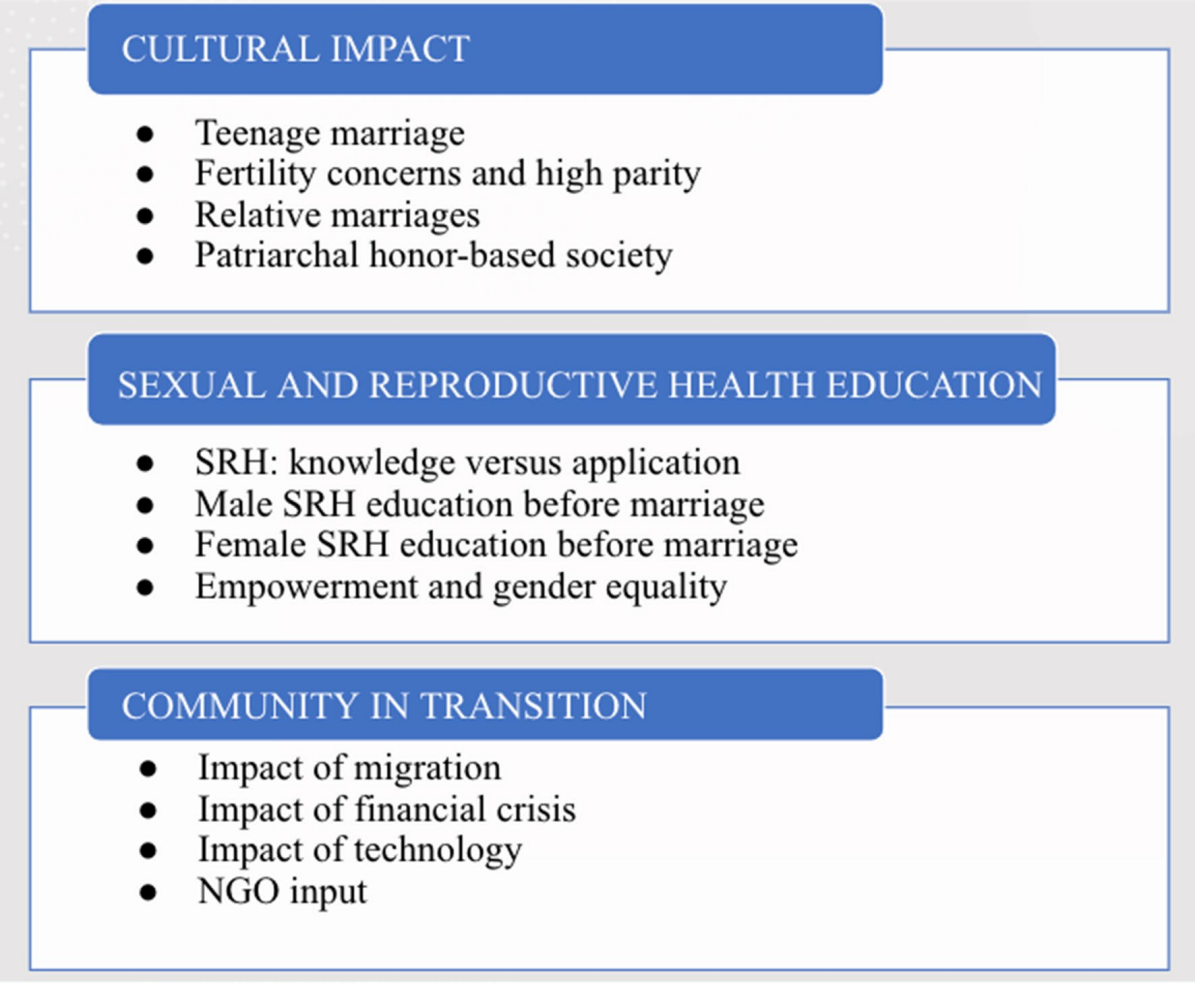
Qualitative Themes and Sub-Themes

### Cultural impact

#### Teenage marriage

**Fig. 4:**
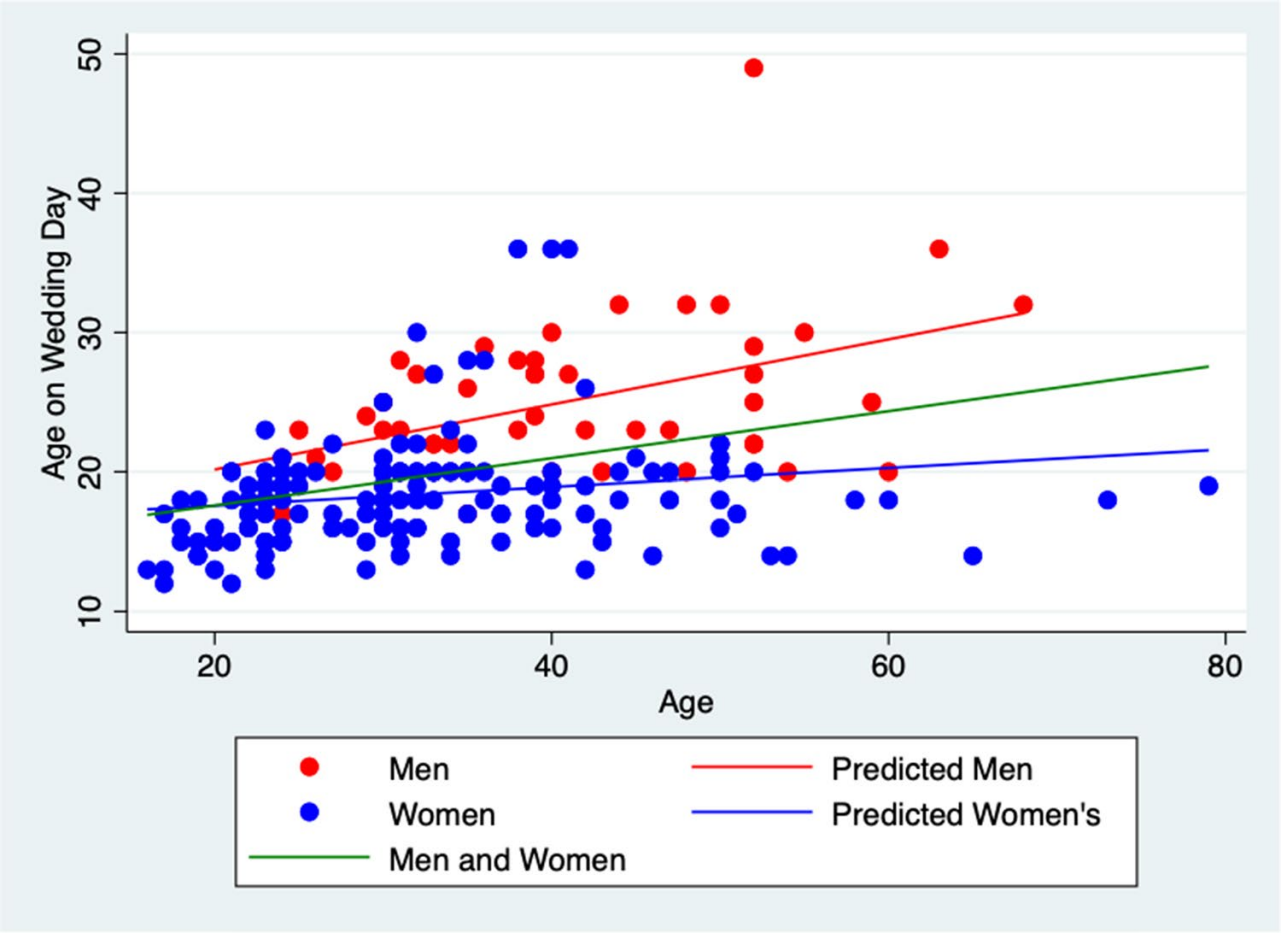
Scatter plot showing age on wedding day by age of participants for men and women

Both women and men in all ITSs identified teenage marriages as common practice saying “*in our culture, even in Syria minor marriages are a very common thing. Everyone, every single girl gets married below 18” (ITS 15 women). A “normal age”* could be as young as *“13, 14, 15” (ITS 13 men) (*Fig. 1*).* Linear regression of age on wedding day by participant age demonstrates a significant fall in average age of marriage of 0.067 (95% CI 0.01- 0.13 SE 0.03) in women, and 0.23 (95% CI 0.10- 0.36 SE0.065) in men across the lifespan of participant (Fig. 4).

Both genders believed that the other encouraged this, with men often saying that women were permitted to express preferences around marriage, and the consensus in 50% of men’s sessions that “*the decision is up to the girls mostly. No one forces anyone here. The girls want to marry early.” (ITS 5 men)* (Table 3)*.* Men in several ITSs also, however, expressed strong preferences for “*fresh young”* girls*, as “that's more desirable" (ITS 5 men)*, and some “*booking”* a fiancée “w*hen the girl is more beautiful than usual, at 13 or 14” (ITS 12 women).* When asked what age was ‘best for girls’ to marry, men in all sessions recognized that this was after the age of 18, but fathers openly disclosed marrying their daughters young, with one saying *"I married my child at 14 and there is nothing wrong with her” (ITS 1 men).* There was only one ITS where both men and women reported that teenage marriage was not practiced (Table 3).

**Table 3:**
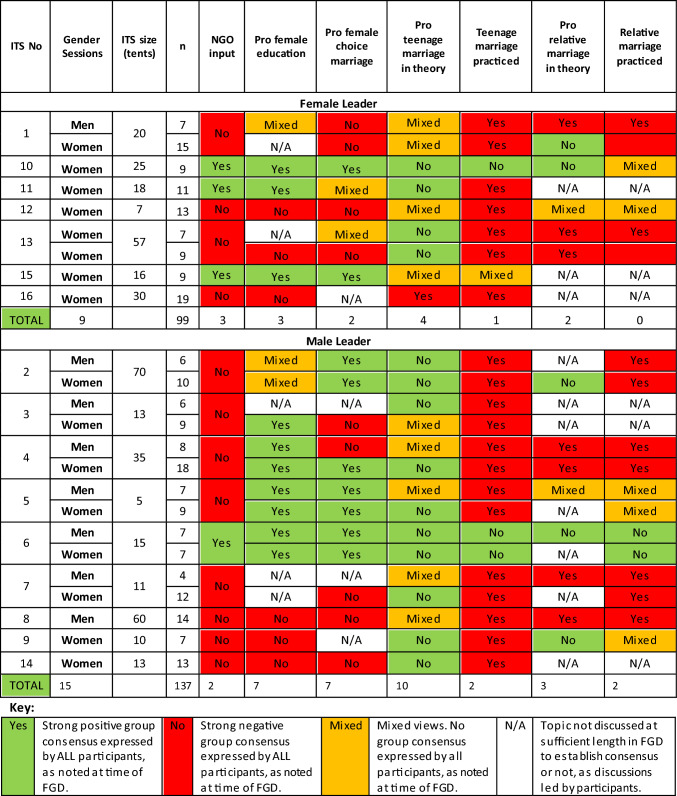
Group consensus in focus group discussions regarding Reproductive & Sexual Health Beliefs in each session, by gender and gender of ITS leader

Women, conversely expressed fears that *"men here only want 14, 15-year-olds, they don't want 20-year-old girls” (ITS 7 women),* and that “*if she reached 20 or 25 the train will miss her. She will not get married anymore” (ITS 12 women).* Interestingly, this manifested in the consensus among women from only six of 13 ITSs being against teenage marriage in theory, including three of 7 ITSs led by women (Table 3). Further, there were only two ITSs where women opposed to teenage marriage stated that they had authority to prevent the practice from taking place, in the other 15 ITSs, even when women disagreed with the practice in theory, they said they could do nothing to prevent it taking place as they “*can't say no to the father” (ITS 3 women)* (Table 3)*.*

A final factor driving teenage marriages expressed by men and women included efforts to protect women from the *“risk for them to bring shame” (ITS 14 women)* on the family, by *“the girl… making mistakes (when she is) excited to be married” (ITS 3 women).* In families in all ITSs, “*everyone feels scared that girls might do something wrong or anything, so that's why the marriages came at early ages” (ITS 1 men).* This is a reason that *“whenever the girl gets her period, she must get married” (ITS 12 women),* as women are “*told by society, ‘Don't let anyone touch you because if [they do touch you, and] your blood comes out of you, you're going to be killed’. Like if she's not a virgin anymore- so that's why they were frightened” (ITS 5 women).* Though there were no reports of girl’s being harmed in this way in the Bekaa, fear of “*shame*” brought on families by women being sexually active by choice, or force, outside marriage was universal. Families felt this particularly acutely considering “*the huge amount of harassment” (ITS 3 women),* though only two individuals discussed this*.*

There was concern in all ITSs that *“marriage under 18 will put the woman in danger… because her vagina is not complete. Her organs aren't complete yet, so she's at risk” (ITS 10 women).* This fear about safety regarding physical health was in some cases paired with concerns about more psychosocial “*problems (girls) face” (ITS 12 women)* if they get married younger. However, overall, regardless of the knowledge in the population around health risks of teenage marriages the sentiment expressed was that “*it’s the tradition here…from our grandparents. It cannot be changed” (ITS 15 women).*

Overall, despite concerns regarding the health risks, pre-existing high rates of teenage marriage are increasing further. This is reportedly now primarily driven by male sexual preference, as it is perceived as a cultural norm. Though women are perceived by men as supporting teenage marriage, this may be due to fear of girls becoming too old to be considered desirable or losing ‘honor’ due to harassment.

#### Fertility concerns and high parity

A significant driver for younger marriages was the value placed on fertility by the entire community. Women were expected to have children immediately after marriage and continue as long as possible “*before the woman ages because if she doesn't have enough children; he'll get married to another woman” (ITS 13 women II).* High parity was a source of pride for families, whereas delivering fewer children brought *“shame on you” (ITS 15 women),* as *“if the woman here doesn't have like 10 children, it will not work out really” (ITS 3 women).*

Another woman said:

*“When someone gets married and is not pregnant after one month, the man will just take her to the doctor or gynecologist. So it's always up to the man. He will marry another woman.” (ITS 1 women).*

In a few cases, women discussed threatened or lived experiences of sexual violence regarding fertility in addition to the threat of divorce or polygamy. One lady said “*that men here actually threaten a woman so if you don't get pregnant after two months of delivery (of another child), I'm going to get married to another woman, or we're going to get divorced. It's very common. Her sister got pregnant after her husband threatened her, and he made her get pregnant by force” (ITS 7 women).*

Pressure to produce children was exerted by husbands, and the husband's family, one lady said that “*when she got married her mother-in-law told her that we only brought you to bring kids, and if you ever take contraception, we're going to make our son get married to another woman” (ITS 13 women).* Mothers-in-law were described by men and women as “*nagging around” (ITS 15 women)* regarding fertility, and pressuring women to deliver more babies. One female participant said.

*“If she obeyed her mother-in-law, she would have delivered 12 children by now”. (ITS 7 women).*

The emphasis on fertility and children in turn, however, was also a driver to reducing teenage marriages, as in getting married later men and women all recognized that a woman would be *“more mature” (ITS 13 men),* and “*her womb is complete, her reproductive systems complete. She might not take risks in being pregnant. She will not give birth early” (ITS 13 women).*

#### Relative marriages

Marriage to close relatives was reportedly practiced in 10 of 11 ITSs where it was discussed (Table 3) despite all participants being aware this increased the risk that *“children will be born with disabilities'' (ITS 1 women)* and cases of this *“happen[ing] in their family” (ITS 1 women) (*Table 3). Men expressed pride in having “*completely*'' healthy families, with “*no divorces… because we married our loved ones” (ITS 1 men)*, saying, “*Everyone here is smart! Our kids are very smart, everyone compliments them, and everyone has good health although we are marrying all our cousins” (ITS 4 men).* In some cases, relative marriages were preferable “*even if the cousin was very bad and the stranger was a good person, they will still choose the cousin… It's culture” (ITS 13 women).*

This practice was reportedly preferred predominantly by *“the father”,* and men in only one of six men’s sessions were unanimously against the practice in theory, and in their ITS (led by a man) the practice had reportedly stopped (Table 3).

In five ITSs, however, though *“it is common, it's getting less, and people are more educated about it. No marriages are happening until full examinations and blood tests are done to see if the genes might cause any problem” (ITS 5 men).* These unspecified blood tests could reportedly detect risk of fetal abnormalities, though no participants knew what was tested. Others said the practice was less common because *"nowadays cousins and relatives feel like the friend zone, the brother zone. They don't feel attracted to each other anymore” (ITS 10 women).*

#### Patriarchal honor-based society

Overall, regarding the timing of marriage, choice of partner, and the timing of children women felt that they “*have no right to decide anything. Everything is decided by the man” (ITS 3 women),* men are “*the boss here” (ITS 11 women)* and a woman must *“do what her husband tells her” (ITS 3 women),* and *“can't say no to the father” (ITS 3 women).* Though the consensus among women in only one of 16 women’s sessions supported teenage marriage in theory, it was practiced in 14/16 of their ITSs (Table 3). Regarding marriage, “*if the boy didn't want to get married, they will not get married, but if the girl's decision is not to get married, she will have to anyways” (ITS 13 women).*

### Sexual and reproductive health education

#### SRH literacy; knowledge versus application

In every session conducted, men and women both identified that the ‘best’ age for girls to marry was “*above 18 above 20 because the girl would be more mature, her organs are complete. Of course. They all agree with that, but nothing applies here. It's the culture here. Everyone gets married at 13, 14. They know it's wrong, but they always keep doing it” (ITS 12 women).*

Further, “*they said that the good time between a child, and another child is two years, three years. That's not applied here. Most of the women don't want to and the decision is up to them. The other half, the decision is up to their husbands and his family” (ITS 4 women).* One woman said “*these three years I have had three kids. They all say two years then above, but they do not apply this” (ITS 13 women).*

Even though “*here since social media… societies here have all the information and everything, [but] nothing is applied here. The application here is zero” (ITS 3 men), “because the people here work upon culture, traditions not on medical studies” (ITS 1 men).*

In some ITS, men were frustrated that “*women here don't care about anything. The men here remind the women to take contraception. They don't care about it; they don't care about vitamins” (ITS 3 men).* Further, *“women don't take vitamins they don't want to. He brought them once for his wife. Two weeks later he found them in the garbage and his wife was pregnant. The doctor prescribed her iron tablets. She isn't taking them she doesn’t want to” (ITS 3 men).* This may demonstrate concerningly low SRH literacy amongst women.

#### Male SRH education before marriage

Men and women deemed it “*obvious” (ITS 12 women)* that men should and did have a thorough understanding of SRH prior to marriage, and “*it's better for men to know [about sex] before marriage… they can help the woman assist her and guide her” (ITS 2 women).* The source of this knowledge was a combination of *"chats between guys” (ITS 12 women), “experience”, “but mostly from the internet and schools” (ITS 5 men).* 10% of men in sessions were unmarried, compared to only 1 unmarried woman participating.

#### Female SRH education before marriage

Historically women were “*forbidden” (ITS 8 men)* to *“know a single thing about sex till the night of their wedding when they actually experience it” (ITS 3 women).* In five women's, but only one men’s session (and only three of 13 sessions in male-led ITSs) participant group consensus was that female education before marriage from any source was *“wrong” (ITS 9 women),* “*doesn't happen [here] and shouldn't happen” (ITS 13 women)* (Table 3). This included *“whenever two women are talking with each other and there's young women beside them, they tell them to go out. We are talking about private stuff” (ITS 3 women).* Women felt “*it's weird for a mother to come and talk with her daughter about this stuff. So they said it's better after the experience that she should find out by herself” (ITS 13 women).* Interestingly, there was group consensus against female SRH education in five of 13 women’s sessions, but only one of six men’s, including significant opposition to SRH education amongst women even in ITS led by women (Table 3).

Arguments against women’s SRH education were that they would “*be more curious about it” (ITS 8 men),* a risk, as *“when someone is deprived of something, and she finds new information and she gets crazy about it” (ITS 14 women).* Men and women felt *“it's not very safe for the girls to know everything” (ITS 16 women)* and *“the more the girl was informed, the more dangerous” (ITS 16 women).*

However, participants acknowledged that women approaching marriage who *“didn't know anything suffered” (ITS 5 men).* Women “*suffered… because they (didn't know anything about contraception) … That's why they have too many children and they don't know what (contraception) is certain, what’s 100%, what’s wise, what’s not. Especially when they were young and newlyweds. That's why they prefer they know” (ITS 4 women).*

Safety was also an argument for women’s education, as “*if the girl doesn't know anything about some topic, she might be curious and want to try it in a different and dangerous way” (ITS 5 women).* The consensus from eight of 13 women’s sessions and five of seven men’s sessions was that despite the *“taboo regarding this stuff” (ITS 12 women)* that they *“would like girls to know that before marriages” (ITS 12 women),* “*as long as it’s used for information and awareness it’s good for the girls” (ITS 5 women). *(Table 3)*.* Most communities preferred information to come from “*the mother because she's the safest source” (ITS 1 women)* and girls “*won’t feel too shy to ask and seek any information” (ITS 12 men).* Alternatives included schools, or in sessions from healthcare professionals as better-informed sources and because *“some girls get shy when their mothers start talking about this subject” (ITS 3 women).*

#### Empowerment and gender equality

Participants varied in whether women’s empowerment was good or bad, but unanimously felt that “*knowledge is power” (ITS 6 men),* allowing women to be *“more independent” (ITS 1 women)*, *“more confident” (ITS 9 women),* and that when “*the girl has the power and the decisions… She is free'' (ITS 1 women).* Some women felt “*when a girl gets married at 20, she might be able to do the chores, take care of her family and she will be more powerful." (ITS 14 women)* whereas others felt that “*women should marry younger than 20 because when she grows up, she will be more independent, and she might not get married anymore (ITS 1 women).*

Though some men were strongly against female empowerment, one man said, *“girls should hold this power and education. There's nothing wrong with it and it's not forbidden in our religion” (ITS 1 men).* Another said *“it's good for the girls and for everyone” (ITS 6 men)* (Table 3).

Despite women being elected leaders in seven ITS, there were no significant differences in age of marriage, or childbirth, number of children, or years of schooling attended between male and female led ITS. Further, there was far less of a consensus around topics of women’s education, teenage and relative marriage amongst men and women in these ITS (Table 3).

NGO-led women’s empowerment sessions conducted in four ITS appeared to have a catalytic effect; “*before women didn't see the light outside. They weren't allowed to work. Now due to the organizations, the NGOs, women are allowed to work whatever she wants, to get out of the house whenever she wants and then have her own salary” (ITS 1 women).* In these four ITS, consensus amongst women was reportedly that they*“decided to step as far as far as possible from societies' decisions and take their own decisions” (ITS 15 women)*; supporting female education, female choice in marriage and contraception usage, reducing the birth rate and condemning (and in some cases stopping the practices of) teenage and relative marriages (Table 3). Further, reportedly in these camps *“men have changed, their mentality has changed, so they like it… even men are getting more educated, more mature” (ITS 10 women).*

No participants discussed formal education for women, or its effects on preventing teenage marriage. Further, there was no significant association between age of marriage and years of school completed for men or women.

Overall, it was unquestioned that men should be thoroughly literate surrounding SRH, though there was minimal evidence that their literacy impacted SRH practices. Significant differences existed between and within ITSs regarding women’s SRH education, and the power it could bring, with arguments either way being primarily based around girl’s safety. NGO led sessions aiming to empower women to work, choose when they marry, and make educated decisions around childbearing reportedly catalyzed major changes in practices in these areas.

### Community in transition

#### Impact of migration

Though participants in some ITS said that “*early marriages happen here more among young girls, not in Syria” (ITS 4 women)* and that previously *“girls didn't get married under the age of 22. Neither do men, but due to the war they are scared… that girls might do something wrong, so that's why marriages came at early ages” (ITS 1 men).*

For most, migration was a bigger impetus for cultural change than war; “*everything changed since they came from Syria, especially because they came. If they were in Syria things would not have changed a lot. They came here and social media has appeared, phones, mixed societies.” (ITS 5 women),* “*a lot of traditions have changed since they moved from Syria to here, a lot” (ITS 8 men).* Many participants were from rural Syrian areas, so “*previously in Syria they would just wake up and do chores with their mothers… the girl now has opinion has her own life” (ITS 8 men).* These changes meant “*that some girls are able to go anywhere”,* and increasing numbers are in employment, though this is in part also a product of the financial crisis.

Further, “*the number of children being born, and pregnancy has decreased… people are getting more educated due to the sessions… people are getting out of their zones, the old ancient zones, and getting to know the world.” (ITS 10 women).* This change in ‘zone’ occurred due to increased education, as mentioned, but also as couples were migrating *“away from their families” (ITS 15 women), "far away from the mother-in-law and the father-in-law so there's no one like that nagging around their heads” (ITS 15 women).* Finally, more *“mixed societies”* and “*the distance between people”* changing has meant that relative marriages “*common in Syria here are going down because the distance has changed. Here some people take Lebanese people…” (ITS 10 women)* or “*women from other governorates, like not from the same family, not from the same society.” (ITS 8 men).*

#### Impact of financial crisis

The financial crisis was undoubtedly the cause of the significant reduction in birth rate, participants said that “*it's not like it was before to have like 10 kids. He is from a family of 10 siblings, but he intends to have only two to three kids due to the financial state” (ITS 3 men).* This has meant that participants reported that “*since the beginning of the crisis, like three years ago, everyone is using contraception, everyone” (ITS 13 women II).* Further “*before this crisis in Lebanon, the men used to want so many children, but when the crisis started, they started, they're advising pregnant women to go get an abortion” (ITS 14 women).* Abortions were previously unheard of and remain illegal in Lebanon.

In practice however, questionnaires showed a reduction in use of contraception, with 43.6% participants reported not using any contraception at the time of the study, and over 15% [29] were depending upon counting their menstrual cycles or withdrawing the penis prior to ejaculation. As questionnaires were distributed following discussion, we were not able to explore reasons for this discrepancy, though 19.65% of participants reported no access to contraception. Literacy around contraception was also limited, with one report that even “*men are taking the [contraceptive] pills so they can prevent pregnancy and don't have to budget for more children” (ITS 2 men).*

#### Impact of technology

The introduction of mobile phones and social media, meant that where previously “*women didn't know [about SRH] before marriage, now [with] the mobiles everything is available.” (ITS 16 women).*

Older men complained that “*since girls young girls right now know how to use smartphones, they have access to websites that they can't even get to.” (ITS 8 men),* and older women that *“the smartphones ruined their brain”,* as *“most girls here are getting (SRH) information from the phones which is very wrong” (ITS 14 women)* and *“dangerous” (ITS 16 women).* Some even argued that as “*this generation seems to know everything” (ITS 16 women)* girls should marry even younger, before they learn anything that could endanger them. (Approximate ages of participants making these comments was noted in reflecxive diary following sessions).

Older men and women were far more comfortable and accepting of younger men using the internet for SRH education, though one father said that “*due to the phones and social media and the pornography industry it's better for his children to learn this information from him rather than to learn from websites, as they may think of applying it in a dangerous way” (ITS 12 men).*

#### NGO input

NGOs had previously run educational sessions around women’s rights, teenage marriage and sexual education in 3 female-led ITSs and one male-led ITS. These sessions were reportedly unanimously well received by both men and women in the ITS (Table 3).

Women described groundbreaking changes as a result of sessions, saying.

*“Before, the woman didn't have any rights, didn't have any voice. She was only for giving birth to children and cleaning. Now, they feel that they have rights. Their voices are heard. They are free. Before woman didn't see the light outside, they weren't allowed to work. Now due to the NGOs, women are allowed to work wherever she wants, to get out of the house whenever she wants, and to have her own salary” (ITS 10 women).*

In all four of these ITSs, teenage marriage and relative marriages were reportedly decreasing, and in the male led ITS, these practices had reportedly completely ceased (Table 3). Women said.

*"Decisions are mostly up to the girls due to the organizations. Because when a girl is 15 and she heard in the sessions it's not healthy to get married, when her cousin proposes, she can communicate with other organizations so they can help her to not get married. So, the other NGOs interfere… they love that because the girl has the power and the choice.” (ITS 10 women).*

Where previously women “*didn't know [about SRH] before getting married, not even their mothers told them anything, they didn't know anything”,* women who had attended sessions themselves said *“they're talking with their daughters, they're making them attend sessions to get more educated” (ITS 11 women).*

Changes were supported by men in the ITS, who said “*all the women in this camp [ITS] work at KAFA organization and they have constant sessions… so you can say that the power here is with the woman, not the men, he said that they only don't know the medical side effects (of contraception), so they would like to know.” (ITS 6 men).* Women said that “*husbands now, they're really flexible and open minded”, “they’re chatting with their partners. They are negotiating” ITS 15 women).*

## Discussion

### Summary

This is the first study authors are aware of to explore the SRH beliefs and practices amongst Syrian men. Findings present changes to SRH beliefs and practices following a decade of displacement and progressive erosion of education and healthcare services aggravated in recent years by the economic crisis in Lebanon.

SRH beliefs remain deeply rooted in a historic, patriarchal culture, celebrating masculinity expressed through fertility and honor (not based upon religion) [6, 9–16, 21, 25, 37]. The protection of women is prioritized, which is perceived, in most cases, to include marrying women as teenagers (while their ‘al sutra’, or honor is intact), marriage to “known” close relatives, and withholding sexual and reproductive education from unmarried women as education may put them at risk [10, 19, 26, 27, 37].

Rapid, drastic and ongoing transitions as a result of war, migration from rural to urban settings, financial crises and increasing contact with more liberal Lebanese societies, technology, and NGOs have, however, resulted in sharp contrasts in beliefs, desires and practices within and between individuals and entire ITSs, and inconsistencies between beliefs expressed and current practices.

### Key findings compared to other studies

#### Marriage

The study population predominantly originated from rural, more economically deprived regions with pre-existing high rates of child marriage, as described elsewhere in the literature [6]. Fear instilled by war, displacement, poverty and worsening political and financial instability resulted in a well-documented further fall in the age of marriages, again demonstrated in this study [6–11, 13–15]. However, we found what could be a significant shift in collective consciousness; with both genders reportedly convinced that early marriage was a cultural norm driven by the other, rather than a choice made by them in view of their current living conditions, or out of fear of assault and harassment. Consensus amongst male participants seemed was that women wanted to marry young, and female participants stated it was primarily due to men’s sexual preferences. Both, however, discussed the importance of protecting women’s honor until marriage, though there was no mention of ‘shame’ regarding the sexual behaviors or SRH knowledge of unmarried men.

Previously, men are reported as associating teenage marriages to financial security or safety, and women linking it to protection from harassment [12, 19, 26]. Education has previously been described as a key protective factor [14]. Instead, our findings could reflect that the practice has far more become embedded as a cultural norm over the ten-year displacement period, despite all participants agreeing on principle that marrying over the age of 18 to 20 was optimal for women’s SRH. Further, the absence of even a discussion about women’s education delaying marriage could demonstrate the true impact of a ten-year atrophy of educational provision.

#### Reduced birth rate

Our novel findings demonstrate cultural shifts necessitated by the financial crisis; women are required to work to support families and the birth rate is reduced in most communities, as families could not afford so many children. Though the birth rate varied between ITSs, male and female participants unanimously felt forced to attempt to reduce the birth rate through either contraception or by seeking illegal abortion (though no participants described succeeding in accessing this yet). Significant cultural and familial pressure remains to conceive immediately after marriage, regardless of the age of the bride. Older Syrian women are drivers of the high birth rate, and couples displaced far from parents-in-law appeared more successful in reducing their family size [20]. Though there have been increased reports of polygamy since leaving Syria, and multiple women described threats of this if they could or would not conceive, no participants reported having more than one spouse [20].

#### Contraception usage

Questionnaire findings demonstrated discussion and shared decision making between spouses regarding contraception, although no male, and few female participants felt that women should have autonomy over contraception. The IUD was the most popular contraceptive, followed by CPs, consistent with other studies and Syrian national statistics, but rates of contraception usage were up to 20% lower than previously reported, and the majority of individuals who would consider an IUD ‘best’ cannot access one [10, 13, 16, 18]. Though not discussed in our study, barriers to contraception include cost, distance (and safe and affordable transportation) and availability, demonstrating the failure in Lebanon to successfully implement even the IAWG minimum initial service package (MISP), which should include free and accessible contraception for displaced women [16, 21].

We found low health literacy, misinformation and taboos around contraception in all sessions making the widely recognized cultural dependence on mothers as the “safest” source of SRH education problematic [12, 26]. Further, given that results demonstrate that men are inextricably involved in women’s contraceptive choices, it is paramount that accurate education is provided to men and women to empower couples to make informed choices. Though men’s SRH knowledge appears to be encouraged universally, there is a lack in provision or accessibility of accurate, reliable, culturally specific material available to men or women on other key SRH topics.

#### Masculinity, SGBV and empowerment

Displacement from a homeland, inability to work due to discrimination, and a resultant perceived loss of respect at being unable to ‘provide’ for their families is known to trigger a sense of emasculation in refugee men as they are stripped of their gendered societal ‘role’ [5, 37]. Frustration due to this emasculation has been linked to increased DV and IPV in other refugee populations, both phenomenon described by multiple women unprompted in our study, though we could not explore this in depth [12, 38]. The forced reduction in birth rate in this isolated population due to increasing financial insecurity may add fuel to this existing fire, as fertility is culturally a core expression of masculinity [20, 38].

This disempowerment of men, however, could be perceived as an opportunity to constructively challenge perceptions of masculinity. A successful example of this was a 12-week program by Veale et al. aiming to provide Syrian men with culturally specific education sessions exploring child development and fatherhood, but also a platform to express feelings and challenge their perception of masculinity [38]. The success of this study, and our own, demonstrate willingness amongst men, despite previous assumptions, to participate in educational activities aimed to empower and better their communities.

We argue that it is vital that men are provided with culturally sensitive, accurate information around SRH, and child health both to address the systematic disempowerment of women currently occurring though teenage marriage and loss of education, but also to accompany ongoing work conducted by NGOs already empowering women through education, advocacy and employment opportunities [19].

### Strengths and weaknesses

This study was conducted on a small budget, with highly limited time and resources in an extremely unstable and challenging setting, as is often the case in the humanitarian sector. As such, though there are many new discoveries and valuable findings, it is limited by weaknesses in study design due to setting specific challenges, which we discuss at length to better inform future work.

#### Study team and positionality

Authors worked in partnership from conceptualization to dissemination with EMA’s Syrian practitioners in Lebanon. Their guidance and familiarity with the populations was the primary reason that recruitment was successful and enabled us to deliver culturally specific SRH information on complex, taboo-ed topics despite the lack of recent, published standardized resources relevant to this population. We used a local Lebanese female interpreter with in-depth knowledge of the setting and culture, as a Syrian interpreter would have been at risk of stigma due to the countercultural content delivered regarding taboo topics. This, however, added complexity to some session dynamics due to the instilled prejudice and animosity between local Syrian and Lebanese populations, though never to a degree affecting the success of the session [5].

AG’s positionality as a female British doctor, conducting sessions asking questions and delivering health messages far from existing cultural norms as an ‘outsider’ to the population was a strength, enabling her to do with minimal fear of stigma. Though introduced as being ‘associated’ with EMA, it was emphasized that participation would not affect access to healthcare, and no incentive was provided to participate besides information shared. Information was presented as ‘the medical perspective’, often as the minority viewpoint in the group discussion conducted within participants homes, but with the authority of being delivered by a medical doctor. In these ways, alongside simple considerations such as seating (on the floor in a circle with participants), and attire, we attempted to flatten traditional hierarchies between researcher and participant and facilitate safe, sensitive spaces to share knowledge, lived experiences and beliefs in a discussion which could be shaped by these [39, 40].

Being female permitted AG to conduct FGDs with male and female participants (likely impossible for a male), though it is probable that this impacted findings compared to if male sessions had been conducted by a male in a such a patriarchal society [39].

Overall, though this specific team was a significant strength of this study, it also presents challenges to reproducing it.

#### Recruitment and study design

ITS leaders welcomed the prospect of running sessions for men and women, though their involvement in the recruitment process undoubtedly impacted the demographics of participants. For example, men only agreed to participate in one of seven ITS led by women compared to seven out of nine male led ITS (Table 3). In some cases, female leaders did not feel empowered to recruit men, but in others men were reportedly away working during daylight hours, or had entirely migrated elsewhere in search of work. There were no ITS where recruitment of married women was not possible. Further, using ITS leaders to recruit risked coercion bias, though we minimized this by emphasizing at the start of each session that participants were free to leave at any point during the session, from the beginning.

Due to the recruitment method, we do not know how many individuals refused to attend sessions, but once gathered, no participants dropped out of sessions (women occasionally left early to attend to their children, as although infants were permitted in sessions, participants preferred to leave them outside in the care of others. We were unable to provide childcare for the duration of sessions). Though involving ITS leaders and communities in recruitment protected participants from stigma and meant that only individuals for whom the session was deemed culturally acceptable to attend did, this process resulted in 91.9% of recruited participants being married. This also removed our control of group size, and as resources and difficulty in accessing camps limited our capacity to run multiple sessions in each camp, and we chose not to deprive any individuals selected of the chance to participate.

Each cohort contained participants with a wide age range, which may have impacted individual’s comfort or ability to discuss sensitive topics, compounded at times by large group size. As cohorts were predominantly exclusively married individuals, participants appeared to feel comfortable in sharing their experiences and views. Further, this recruitment method minimized the stigma of attending as decisions to attend were taken collectively, not selected by us or individually. Content covered was known by the entire cohort, as opposed to interviews which could have exposed individuals to stigma based on participation [29]. FGDs enable participants to share knowledge, lived experiences and beliefs in a setting, which can then be shaped by these, thus flattening traditional hierarchies between researcher and participant, though in a study focusing on sensitive topics, this inherently risks bias due to social desirability (40, 41). In an exploratory study the freedom of expression offered by a focus group was vital, as we wanted participants to be empowered to direct topics to align with their priorities and concerns. We were encouraged that the impact of any social desirability bias was minimal by the frequently contrasting views expressed between, and within, FGDs.

#### Data quality and accuracy

FGD audio recordings created in crowded tents were not of sufficient quality to transcribe each participant verbatim in Arabic, leaving us dependent on transcription of our interpreters' quotes. These were checked rigorously for precision and accuracy by the study team, where recording quality permitted, to ensure that findings are as representative as possible of the population. We also elected to include analysis of ‘group consensus’ amongst participants in FGDs, as noted in the reflecxive diary at time of FGDs. We felt analysis of this was important as it enabled us to present novel findings around contrasts between female and male ITS, and male and female participants on topics which we were not able to cover in questionnaires due to cultural sensitivity. Though there is a limit to the strength of any conclusions that can be drawn from this - opinions expressed by individuals might be impacted by more dominant participants, or cultural norms or taboos - these limitations are arguably common to all conclusions drawn from FGD settings. Finally, the combination of conducting FGDs, followed by confidential questionnaires, resulted in disparities in data around beliefs expressed in FGD and practices in the questionnaires which we could not explore further. Ongoing work is needed to explore reasons for these, particularly around contraception use.

### Conclusions and recommendations

Ultimately, SRH is a complex, multifaceted and contested area in this vulnerable, hard-to-reach population, governed to a large degree by systemic issues around the policies determining Syrian access to care, employment and education in Lebanon. This study demonstrates the impact of a decade of depleted education and employment prospects on an existing socioeconomically disadvantaged, and culturally conservative, population. Groundbreaking, holistic change is required to tackle systemic issues, particularly to re-establish the narrative around female education and opportunity.

Despite this, this study is a small but highly important proof-of-concept study highlighting the possibility, and importance, of engaging men in SRH education and discussion. This is paramount to truly empower women when working in such a heavily patriarchal culture, and disrupt intergenerational cycles of SGBV. We also highlight the importance of providing safe spaces and access to SRH, and SRH education for women alongside demonstrating the exciting possibility for this to be through culturally specific online resources.

## Data Availability

Dataset available on request from the corresponding author. In process of sharing in online depository as per university guidelines.

## Appendix 1: Topic frame for FGDs

1. Description of basic female reproductive anatomy and the menstrual cycle with visual aids & discussion of female hygiene.
2. Discussion around age of marriage in each community, age of childbearing, and time interval between pregnancies
3. Information (WHO guidance) regarding health risks of teen marriage and pregnancy, alongside recommended time interval between children, with reasons why.
4. Discussion around relative marriages. If prevalent information regarding health risks.
5. Basic information about the widely available forms of contraception
6. Answer any questions about contraception.
7. Discussed perceptions of SRH education for married, and unmarried men and women.
